# Biomimetics on the micro- and nanoscale – The 25th anniversary of the lotus effect

**DOI:** 10.3762/bjnano.14.69

**Published:** 2023-08-03

**Authors:** Matthias Mail, Kerstin Koch, Thomas Speck, William M Megill, Stanislav N Gorb

**Affiliations:** 1 Institute of Nanotechnology (INT), Karlsruhe Institute of Technology (KIT), Hermann-von-Helmholtz-Platz 1, D-76344 Eggenstein-Leopoldshafen, Germany; 2 Faculty of Life Sciences, Rhine-Waal University of Applied Sciences, D-47533 Kleve, Germany; 3 Plant Biomechanics Group, Botanic Garden, Faculty of Biology, University of Freiburg, Schänzlestrasse 1, D-79104 Freiburg, Germany; 4 FIT, Freiburg Center for Interactive Materials and Bioinspired Technologies, Georges-Köhler-Allee 105, D-79110 Freiburg, Germany; 5 FMF, Freiburg Materials Research Center, Stefan-Meier-Strasse 21, D-79104 Freiburg, Germany; 6 Centre for Biomimetic and Natural Technologies, Faculty of Technology and Bionics, Rhine-Waal University of Applied Sciences, Marie-Curie-Str. 1, D-47533 Kleve, Germany; 7 Department of Functional Morphology and Biomechanics, Institute of Zoology, Christian-Albrechts-University of Kiel, Am Botanischen Garten 1–9, D-24118 Kiel, Germany

**Keywords:** biomimetic surfaces, hydrophobicity, lotus effect, Salvinia effect, superhydrophobicity, wettability

**Figure 1:**
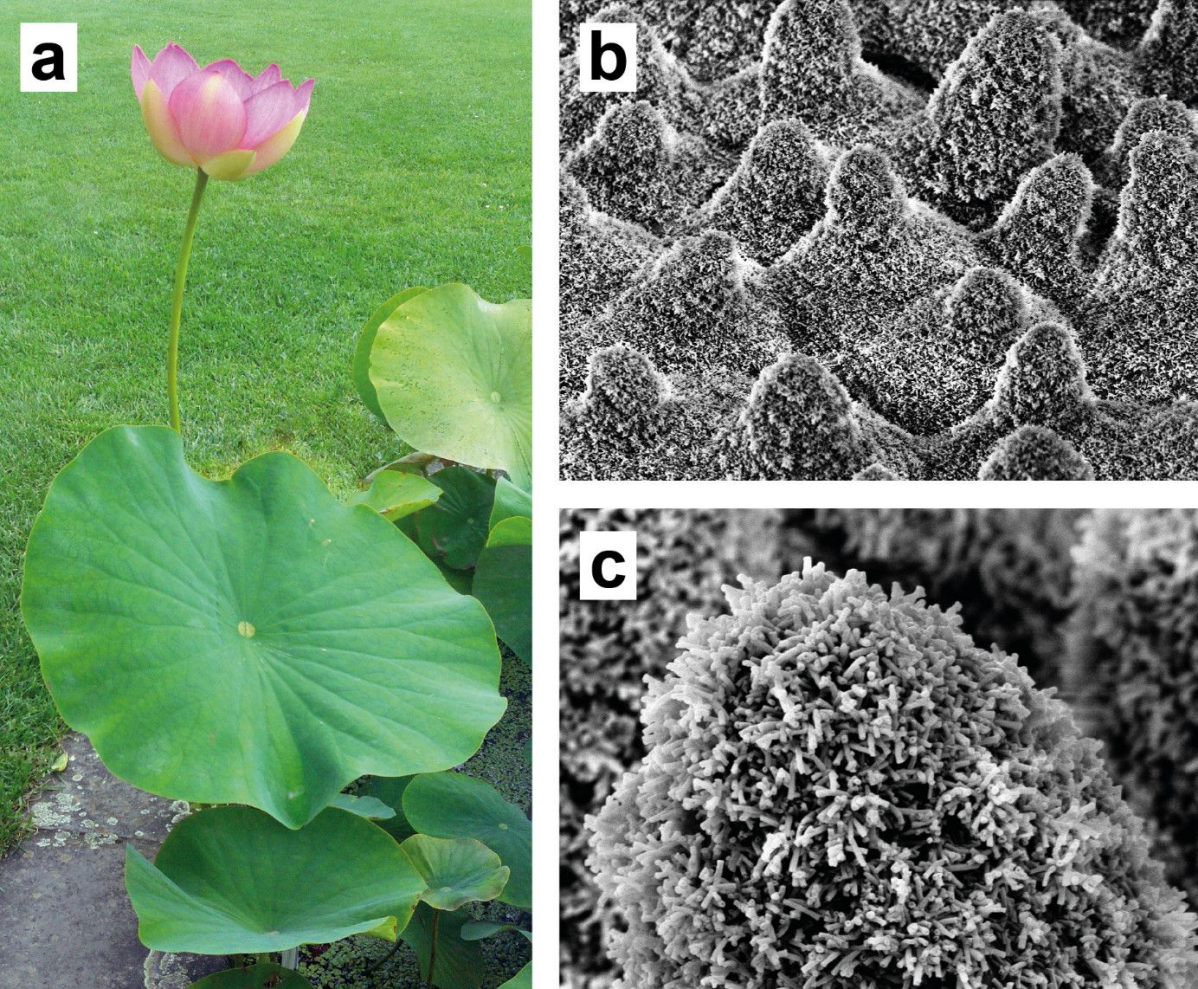
Biological archetype and eponym of the lotus effect: The sacred lotus (*Nelumbo nucifera*). a) Photo of a lotus plant. b) Scanning electron microscopy (SEM) image of the hierarchically structured surface of a lotus leaf. The first level of the surface structure consists of papillae formed by epidermal cells with a height and diameter of several microns. c) This structure is covered by wax crystals, leading to the extreme superhydrophobicity of the surface.

In 1997, Wilhelm Barthlott and Christoph Neinhuis published the paper “Purity of the sacred lotus” [1] in which they described the superhydrophobic surfaces and the self-cleaning ability of some plants (the so-called “lotus effect”, see Figure 1). This paper led to a paradigm shift in surface sciences. It generated a lot of interest at the time and continues today to inspire numerous publications and technical developments [2]. Although the phenomenon of the outstanding water repellence of some plant surfaces had been known for over 2000 years, the functional principle behind it and the detailed physicochemistry of superhydrophobic biological surfaces had remained unexplored [3]. Uncovering this water repellence mechanism led to a revolution in the development of such surfaces, which promise huge potential for inspiration for technical innovations and applications.

In 2022, we celebrated the 25th anniversary of this significant botanical publication, which was an important milestone for the field of biomimetics. We, the guest editors of this thematic issue, could imagine no better way to celebrate that achievement than to gather a new set of publications in the fields of biology and materials science. In fact, these publications identify current topics in surface biomimetics and provide an overview of the development of superhydrophobic and self-cleaning biological and bioinspired surfaces over the past 25 years.

The field that today is called “biomimetics” is almost as old as human history. Many examples in the literature show that in the early days of humankind the concept of “copying” nature or learning from it arose (e.g., the story of Daedalus and Icarus [4]). This and other examples show that often physical phenomena in animals and plants can be described; however, it is not possible to immediately understand the mechanisms behind them and to transfer those to actual applications. This was also the case with the lotus effect.

Biomimetics was finally given a name in the second half of the last century, interestingly just as humankind was finally achieving Icarus’s dream of flying closer to the sun. The big push in the field came with the rapid development of new research techniques and methodologies that enabled detailed investigation of biological archetypes. High-detail microscopy, analysis systems, and novel simulation tools helped to decode the secrets of nature, while new fabrication techniques helped to transfer these findings into technical applications. In the last decades, production methods have enabled the transfer of many outstanding properties of the biological archetypes into innovative biomimetic products at reasonable cost.

These developments helped to bring the field of biomimetics forward, initially by fostering interest in biomimetic approaches, and finally by establishing biomimetics as its own field of science. In this thematic issue, we have gathered 15 publications from several fields of interest, showing the huge diversity of biomimetic approaches.

Surapaneni et al. [5] present in the paper “Polarity in cuticular ridge development and insect attachment on leaf surfaces of *Schismatoglottis calyptrata* (Araceae)” a study of the development of cuticular ridges on the adaxial leaf surfaces during leaf ontogeny of the tropical Araceae *S. calyptrata*. The structure of these microscopic ridges helps plants to defend themselves against pest insects by reducing the frictional forces experienced when they walk on the leaves. This structure might also provide mechanical stability to the growing plant organs and has an impact on the wettability of the leaves. Using polymer replicas of adaxial leaf surfaces at various scales, the surface structure was quantitatively described with confocal laser scanning microscopy and a surface analysis software. The data show a polar development of cuticular ridges and a basipetal ridge progression during leaf ontogeny. Traction experiments with Colorado potato beetles as model species showed low walking frictional forces on both, freshly unrolled leaves as well as adult leaves. These results extend the understanding of the mechanisms used by plants to defend themselves against herbivorous insects by changes in the leaf morphology on the macro- and microscale.

Li et al. [6], in the paper “Effect of sample treatment on the elastic modulus of locust cuticle obtained by nanoindentation”, investigate the mechanical properties of the cuticle that builds the surface of insects and related groups of animals. The cuticle is one of the most abundant, but least studied biological composites. In their study, the authors use a nanoindentation technique to investigate the effect of freezing, desiccation, and rehydration on the elastic modulus of the hind tibial cuticle of locusts. All of the treatments significantly influenced the mechanical properties of the latter.

Gorb et al. [7], in the paper “Hierachical epicuticular wax coverage on leaves of *Deschampsia antarctica* as a possible adaptation to severe environmental conditions”, used cryo-scanning electron microscopy to study surfaces of *D. antarctica*, one of the only two flowering plants native to Antarctica. The results show that the two-layered wax, which densely covers both leaf surfaces, contributes to the plant's adaptation to severe environmental conditions in Antarctica by increasing its resistance to cold temperatures, icing, harmful UV radiation, and dehydration.

In the paper “Micro-structures, nanomechanical properties and flight performance of three beetles with different folding ratios”, Sun et al. [8] use modern, high-precision instruments to uncover the relationship between wing morphology and flight performance of three species of beetles. They use a high-speed camera to track flapping frequency, quantify the surface geometry of the wings with a super depth-of-field microscope, measure cross-sections of veins of wings via SEM, and characterize the nanomechanical structural and elastic behaviour of the structures using a nanoindenter. The authors use the detailed observations obtained to explore the relative aerodynamical performance of the three species of beetles flying tethered in a wind tunnel. The results show that at low wind speeds, typical during insect flight, the species with the highest folding ratio and highest flapping frequencies produced the highest lift-to-drag ratio. The results are in agreement with other studies of flapping insects with high aspect-ratio wings. The authors then relate the aerodynamic performance of the three species studied to the behavioural ecology requirements of their niches to generate exciting avenues of bioinspiration for flapping micro-air-vehicles.

Bargel et al. [9] discuss in their review paper “Bioselectivity of silk protein-based materials and their bio-inspired applications” the importance of tailoring bioselective, biologically active, and multifunctional materials for biomedical applications in biomaterial research. The review was focused on two major topics, the first one being biological processes and surface interactions involved in the bioselective adhesion of mammalian cells. The second topic of the review was on repellence of microbes on protein-based material surfaces, highlighting the importance of materials made of recombinant spider silk proteins. Biomaterials that stimulate and interact with cell receptors to support binding and subsequent physiological responses of multicellular systems have attracted much interest in the last years. This holds especially true for materials that exhibit microbial repellence or antimicrobial behaviour to reduce inflammation, while at the same time selectively enhance regeneration in host tissues. The authors point out that in this context, protein-based materials and especially silk materials are interesting candidates due to their natural origin, biological activity, and structural properties. These exciting recombinant production technologies allow for application-specific modification to develop adjustable, bioactive materials as shown in this review article.

In the paper "Design of a biomimetic, small-scale artificial leaf surface for the study of environmental interactions" by Huth et al. [10], the authors developed wax-coated artificial leaf surfaces with chemical composition and wettability of wheat (*Triticum aestivum*) leaves. Such artificial leaves are of interest for in vitro studies of interactions of plant surfaces with living organisms and the non-living environment, as demonstrated by Huth et al. [11], where the artificial leaves were used to investigate the influence of wax chemistry and surface wettability on the development of *Blumeria graminis*, the pathogenic wheat powdery mildew.

In “Interaction between honeybee mandibles and propolis”, Saccardi et al. [12] report on the honeybee propolis, a substance used by bees to seal their hive and protect the colony against pathogens. Since propolis is quite a sticky substance, the authors analysed whether the mandibles of the bees show specific anti-adhesive properties, enabling them to manipulate the propolis. Adhesion experiments with propolis and bee mandibles were performed and the mandibles were analysed using (cryo-) scanning electron microscopy, indicating that a fluid substance covers the medial surface of the mandibles reducing propolis adhesion.

Weiser et al. [13] take biomimetics into industrial production with their paper “Roll-to-roll fabrication of superhydrophobic pads covered with nanofur for the efficient clean-up of oil spills”. Nanofurs are densely packed hairy surfaces which demonstrate superhydrophobicity, functioning similarly to the lotus leaf whose science we are celebrating with this thematic issue. As with many biomimetic inventions, the complexity of their structure which gives them their properties also makes them difficult to manufacture. Nanofurs are created relatively easily in a lab environment; however, a durable, environmentally friendly, cost-effective, and large-scale industrial production has been elusive so far. The authors present a new technique that has the potential to achieve the breakthrough the researchers in the field have been searching for. This technique uses a typical industrial process in which a continuous film is run over and between rollers. The so-called roll-to-roll process takes the material through a few steps. In the first step, a molten polypropylene layer is laminated onto a carrier film and run over rollers to cool down. Then the combined laminate is forced through a gap between a hot sandblasted roller and a cold smooth one. The surface roughness and temperature difference create the hairy structured surface on the polypropylene side. In the final step, the structured film is separated from the carrier. The authors were able to set the hair length and density with the usual industrial control variables of speed, temperature, and gap width. The obtained result was a very satisfactory nearly superhydrophobic material which the authors used to create an effective oil–water separation product they called nanopads. What this paper demonstrates, in addition to the not insignificant contribution it makes to the science of structured surfaces, is that with a bit of creativity and a solid understanding of industrial processes, complex structured biomimetic inventions can indeed be effectively brought to the market.

Lifka et al. [14] stick to the nanofibre and production topics in their paper “Laser-processed antiadhesive bionic combs for handling nanofibers inspired by nanostructures on the legs of cribellate spiders”. Here the challenge is to handle nanofibres which naturally stick to surfaces due to the van der Waals energy of surface interaction. Spiders which regularly process nanofibres into silk have evolved a structure on the surface of their hind legs to which the nanofibres do not stick. The authors use the geometry of the spider system to develop an elegant mathematical model of the interaction between the fibres and the surface. They then test their predictions using a structured metal mimic of the spider legs. They find that for some metals, in which they were able to generate surface geometries that corresponded to the boundary conditions of their model, the peel-off force is lower over the structured surface than over a polished one. Their understanding of the relationship between the surface structure and the peel-off force will now make it possible to tune and then scale up the fabrication of tools to handle nanofibres in industrial processes.

Fibre–surface interactions are also the main theme in “Growing up in a rough world: scaling of frictional adhesion and morphology of the Tokay gecko (*Gekko gecko*)” by Cobos and Higham [15]. In the excitement of the biomimetic abstraction and development process, in particular when it looks like it is leading to a product, one can sometimes miss some of the important secondary features of the biological archetypes. This paper reminds us of this as it explores the relationship of gecko foot pad adhesion and the geometry of the setae with age and size of the animals. The authors find that the diameter of the fibres and their density in the toepad do not change with size in this species. The toepads scale isometrically with body size, and the setae get relatively shorter with body size. The scaling is also reflected in the adhesive forces the animals are able to produce on surfaces of varying roughness. These observations make specific predictions about the behavioural ecology of this species in the wild. They also remind us to keep looking at the archetypes and closely related species for further inspiration as we develop our biomimetic abstractions into actual products.

This theme is picked up again by Konrad et al [16] who describe their nonlinear journey through bioinspiration in “Straight roads into nowhere – obvious and not-so-obvious biological models for ferrophobic surfaces”. The challenge to be solved was to protect copper air delivery tubes from molten iron during the smelting process. The initial concept was to maintain a layer of air between the vulnerable copper surface and the molten iron. The obvious archetypes, *Salvinia* and *Collemba*, which do something similar in their natural environments, were selected as likely sources of bioinspiration. It quickly became obvious that the intricate physics of both archetypes was not appropriate for the industrial conditions of the steel oven. The project would have stopped there had it not been for the combined knowledge of other biological processes by the authors. They looked to a seemingly unrelated archetype, the xylem system of trees, which uses pits instead of extrusions to create its surface properties. This led the team to a new approach and eventually to a successful product. The authors present the case study as a reminder that the breadth of biological knowledge is still the foundation on which biomimetics is built.

The paper by Mail et al. [17] “Dry under water: air retaining properties of large-scale elastomer foils covered with mushroom-shaped surface microstructures” focuses on superhydrophobic surfaces, not only on water repellency, but also on the capability of some surfaces to keep stable air layers under water – the so-called Salvinia Effect. Such air layers are of great importance for drag reduction (passive air lubrication), antifouling, sensor applications, or oil–water separation. Up to now, based on the investigations of some biological role models (e.g., the floating fern *Salvinia* or the backswimmer *Notonecta*), several prototypes of such surfaces have been developed. In this publication, the authors analyse a novel biomimetic surface, which was initially developed for a different purpose, for its air retaining properties: an adhesive elastomeric film with mushroom-shaped surface microstructures that mimic the adhesion system of animals. They show that this elastomer foil provides good air retention capabilities and is a promising material for the development of an economically and efficient biomimetic air retaining surface, which could be produced on large scales. Furthermore, this publication shows some nice examples of the multi-functionality of some biological surfaces as well as of some biomimetic developments.

Rounding out the thematic issue and looking into the future, Leivas and Barbosa [18] present their model for “Atmospheric water harvesting using functionalized carbon nanocones”. The challenge of harvesting water directly from air of varying humidity is a tantalizing one. There are several biological examples in both the animal and plant kingdoms which could serve as archetypes for dehumidifiers or water purifiers. The authors here present a model based on a carbon nanotube structure that might finally provide a route to the breakthrough in this field. Their archetype, the Namibian desert beetle, uses a geometric separation of hydrophilic and hydrophobic regions to harvest and drive water along its body. The authors built on this inspiration to develop carbon nanotube cones which similarly separate the regions. Their model predicts the specific geometry and surface properties that will be required to create a low-pressure system for harvesting water vapour.

Rebora et al. [19], in the paper “The origin of black and white coloration of the Asian tiger mosquito *Aedes albopictus* (Diptera, Culicidae)” analysed the reflectance spectra of the white scales as well as the micro- and nanostructure of the black and white scales on the tarsi of *A. albopictus*. The results show that the white colour is produced by the hierarchical micro- and nanostructure of the surface. The reflection of light depends on the angle of incidence. In addition to superhydrophobicity, these results suggest that the scales have another function – their hierarchical structures produce the brilliant white colour, and therefore the surface is multifunctional.

In the paper “Suspension feeding in Copepoda (Crustacea) – a numerical model of setae acting in concert”, Filippov et al. [20] present a numerical modelling approach to understand the influence of different parameters on the feeding efficiency of suspension feeding in common Crustacea. The results show that a system combining long and short setae with different mechanical properties has the best performance. The numerical simulation can in future be easily adapted to other systems and therefore be used for biomimetic developments (e.g., in the field of filtration technologies).

**Figure 2:**
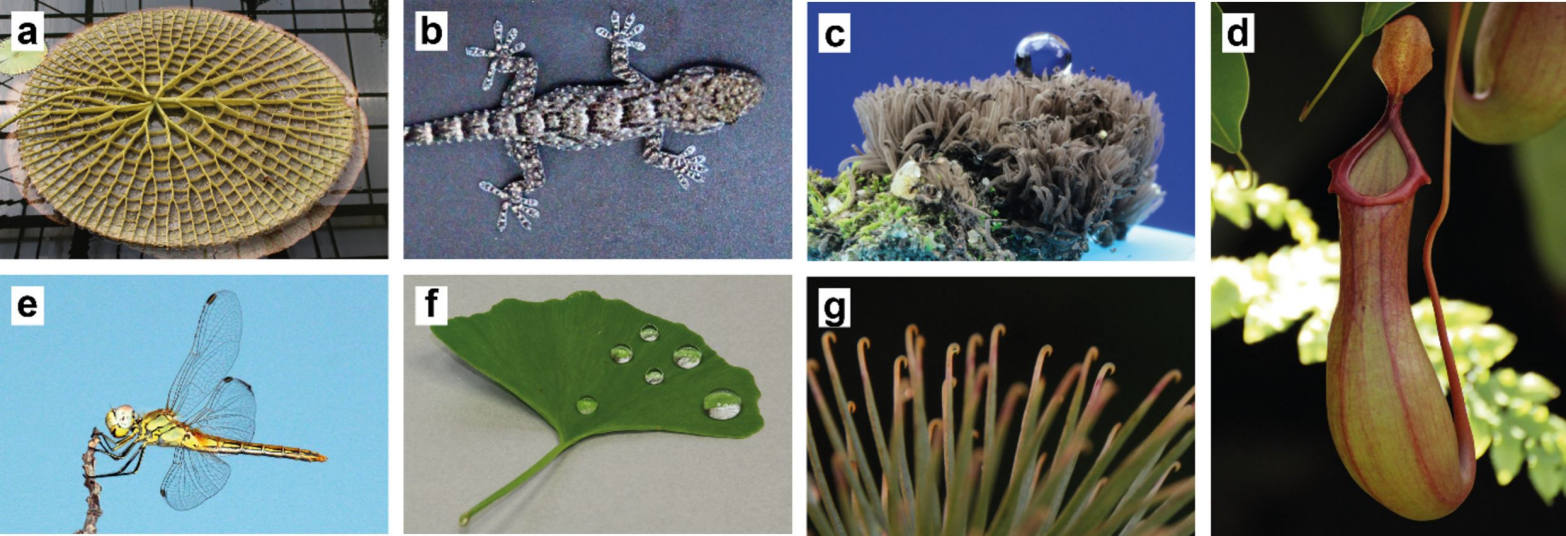
Biological archetypes – The photographs show just a few examples of the incredible diversity of “biological prototypes” nature provides, which enable the development of biomimetic solutions on a brought spectra of different fields of application. a) A floating leaf of a giant water lily (*Victoria*), a nice example of a light-weight structure. b) A gecko – one of the best-known archetypes for biomimetic adhesion. c) An example of the curiosities that nature holds – a slime mould whose spores exhibit superhydrohobic properties. d) A pitcher plant, which has on the edges a special and very slippery surface to prevent insects from climbing out. This surface structure is the opposite of adhesive surfaces. e) A dragonfly. Archetype for robotic applications but also known for their superhydrophobic and transparent wings. f) An example of the early evolution of superhydrophobic surfaces: the leaf of a ginkgo tree. g) Probably one of the best-known examples of biomimetic developments: the greater burdock (*Arctium lappa*) – the archetype for the Velcro tape.

These examples on the one hand handle with biomimetic ideas that are more popular and on the other hand show some extreme cases, where nature provides highly specialized, sophisticated solutions (see Figure 2 for examples). In many cases, nature provides the first solution for a technical problem that has not been solved for a long time. In other cases, the biological systems, and therefore their related biomimetic solutions, often show a much higher efficiency than the majority of all previous non-biomimetic engineering solutions.

The diversity of biomimetic approaches is incredible and the sheer number of topics, projects, and publications gives only a brief idea of how many solutions living nature still can provide – as long as we look carefully and with the right mindset. Considering that current publications roughly estimate a total number of 10.000.000 different species living on our planet and that only about 20% of them are known, one can easily understand the incredible number of new possible findings in the field of biomimetics. This also shows how important it is to protect the environment and biodiversity, as all those organisms in addition to their ecological role in the biosphere are potential biological “prototypes” for technical developments, which might significantly contribute for a “greener future”.

Taking into account the fast development of biomimetics as a research field in recent years, we took the 25th anniversary of the lotus effect publication as the perfect occasion for this thematic issue. The issue continues the reputable tradition of this journal, building on four previous thematic issues with biomimetic topics [21–24]. The publications gathered in the present thematic issue give an idea of the great variety of areas in this exciting field of biomimetics, and many different cases where nature could help us to find solutions for technical problems. By seeing this exciting diversity of ideas, functionalities, and solutions, we are very much looking forward to the developments of this field in the next 25 years.

Matthias Mail, Kerstin Koch, Thomas Speck, William M. Megill and Stanislav N. Gorb

Eggenstein-Leopoldshafen, Kleve, Freiburg, and Kiel, July 2023
